# High-resolution multi-breath-held 3D volumetric T1 mapping acquisition: analysis of volume measurements of small structures using a respiratory motion phantom

**DOI:** 10.1186/1532-429X-17-S1-W23

**Published:** 2015-02-03

**Authors:** Keigo Kawaji, Sui-Cheng Wang, Akiko Tanaka, Hui Wang, Roberto Lang, Amit R Patel

**Affiliations:** 1 Medicine, Section of Cardiology, The University of Chicago, Chicago, IL, USA; 2 Surgery, The University of Chicago, Chicago, IL, USA; 3 Biomedical Engineering, Northwestern University, Evanston, IL, USA; 4 Philips Medical Systems, Cleveland, OH, USA

## Background

Myocardial T1 mapping is performed using 8-10mm thick slices; however, this is unsuited for accurate quantification of small structures. We propose a novel 3D-pulse-sequence with improved through-plane spatial resolution. In this study, we use a respiratory motion phantom to compare the accuracy and precision of volume measurements made using this 3D-sequence against a reference 2D-technique.

## Methods

The proposed 3D-sequence employs a Cartesian projection of radial sectors partitioned by twice the number of total breath-holds. Each opposite sector pair is acquired in a single readout, while k-space center is acquired at the acquisition window midpoint. Partial kz (~62%) and circular shutter Field-of-View (FOV) (~27% reduction) was used to yield ~3x improvement in through-plane resolution.

**Figure 1:**
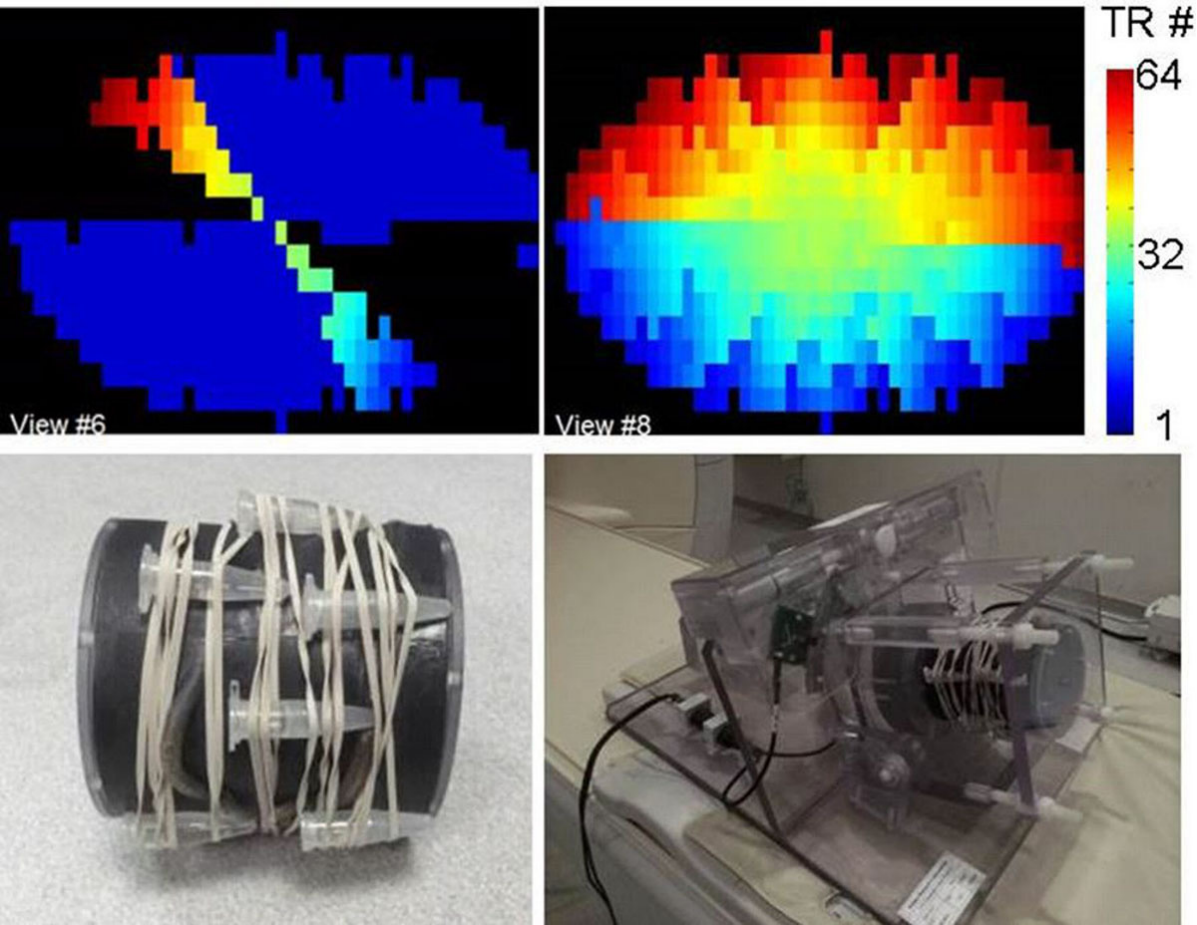
(top row): Profile order of the 3D Acquisition over multiple heart beats. (bottom row): Motion Phantom.

Imaging was performed using 1.5T MRI (Philips Achieva) with a 4-channel array on a respiratory motion phantom with 12 conical vials containing varying gadolinium concentrations diluted in 1cm^3^ (fig 1). A 5-(3s)-3 MOLLI scheme was used for both 2D- and 3D-imaging across the same FOV. The following respiratory patterns were examined: 1) no motion, 2) small (4mm) respiratory shifts, and 3) large (8mm) respiratory shifts. Through-plane registration error was introduced in 2D, while motion blurring was introduced in 3D volumes. 4 vials were partially out of the FOV to test partial volume effect on the techniques. The following parameters were used for both 2D- and 3D-MOLLI: FOV (185x185x80mm), 8 breath-holds, SENSE factor 2, in-plane resolution (1.7x2.1 mm) with 2D vs 3D slice thickness = 10 vs 3.1 mm (8 and 26 slices, respectively). All vials were measured across every depicted slice using both T1-weighted images and calculated T1 maps. T-test (difference) and F-test (variance) were used to compare the 2 techniques.

## Results

**Figure 2:**
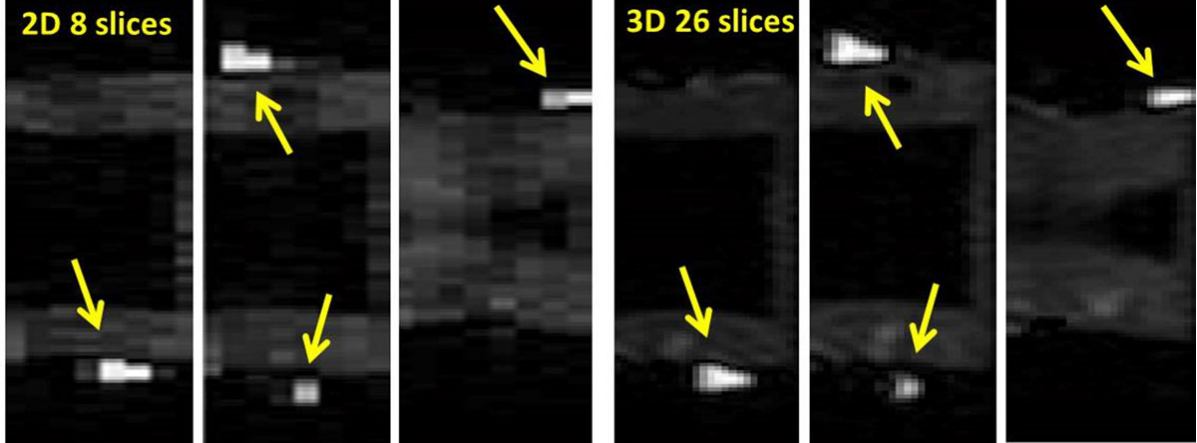
(left): 2D and (right) 3D reformats of the acquired T1 weighted volumes. The conical vials are depicted more clearly in the 3D MOLLI reformats.

The 1cm^3^ volumes were more accurately and precisely measured using 3D in the absence of motion (2D vs 3D: 0.95±0.08 vs 1.02±0.03cm^3^; p<0.05) and in the presence of large respiratory shifts (1.26±0.35 vs 1.01±0.08cm^3^; p<0.005). During small respiratory shifts, both 2D- and 3D-techniques overestimated the vial volumes (1.15±0.12 vs 1.09±0.07cm^3^; p=0.1). Delineation of the vial shape was feasible in 3D but not 2D (fig 2). 2D overestimated the volume of vials incompletely in the FOV, even in the absence of respiratory motion; whereas 3D yielded the expected smaller volumes (2D vs 3D: 1.06 ± 0.20 vs 0.83 ± 0.08 cm^3^).

## Conclusions

The proposed high-resolution 3D-T1-mapping acquisition provided improved volumetric measurements compared to the reference 2D approach under various respiratory motion conditions.

## Funding

N/A.

